# Network Analyses: Understanding the Pathways of Functional Improvement in Schizophrenia

**DOI:** 10.1192/j.eurpsy.2022.115

**Published:** 2022-09-01

**Authors:** A. Mucci, S. Galderisi, P. Rocca, A. Rossi, A. Bertolino, P. Rucci, M. Maj

**Affiliations:** 1 University of Campania Luigi Vanvitelli, Department Of Psychiatry, Naples, Italy; 2 University of Turin, Department Of Neuroscience, Section Of Psychiatry, Turin, Italy; 3 University of L’Aquila, Department Of Biotechnological And Applied Clinicalsciences, Section Of Psychiatry, L’Aquila, Italy; 4 University of Bari Aldo Moro, Basic Medical Science, Neuroscience And Sense Organs, Bari, Italy; 5 University of Bologna, Department Of Biomedical And Neuromotor Sciences, Bologna, Italy

**Keywords:** Recovery, everyday life skills, cognition, functional capacity

## Abstract

Improving real-life functioning is the main goal of the most advanced integrated treatment programs in people with schizophrenia. The Italian Network for Research on Psychoses used network analysis in a four-year follow-up study to test whether the pattern of relationships among illness-related variables, personal resources and context-related factors differed between patients who were classified as recovered at follow-up versus those who did not recover. In a large sample (N=618) of clinically-stable, community-dwelling subjects with schizophrenia, the study demonstrated a considerable stability of the network structure. Functional capacity and everyday life skills had a high betweenness and closeness in the network at both baseline and follow-up, while psychopathological variables remained more peripheral. The network structure and connectivity of non-recovered patients were similar to those observed in the whole sample, but very different from those in recovered subjects, in which we found few connections only. These data strongly suggest that tightly coupled symptoms/dysfunctions tend to maintain each other’s activation, contributing to poor outcome in subjects with schizophrenia. The data suggest that early and integrated treatment plans, targeting variables with high centrality, might prevent the emergence of self-reinforcing networks of symptoms and dysfunctions in people with schizophrenia.

**Disclosure:**

Honoraria, advisory board, or consulting fees from Angelini, Astra Zeneca, Bristol-Myers Squibb, Gedeon Richter Bulgaria, Innova-Pharma, Janssen Pharmaceuticals, Lundbeck, Otsuka, Pfizer, and Pierre Fabre, for services not related to this abstract

